# Ultrastructural characterization of hippocampal inhibitory synapses under resting and stimulated conditions

**DOI:** 10.1186/s13041-024-01151-0

**Published:** 2024-10-22

**Authors:** Jung-Hwa Tao-Cheng, Sandra Lara Moreira, Christine A. Winters

**Affiliations:** 1 NINDS Electron Microscopy Facility, National Institute of Neurological Disorders and Stroke, National Institutes of Health, Bethesda, MD 20892 USA; 2 Laboratory of Neurobiology, National Institute of Neurological Disorders and Stroke, National Institutes of Health, Bethesda, MD 20892 USA

## Abstract

The present study uses electron microscopy to document ultrastructural characteristics of hippocampal GABAergic inhibitory synapses under resting and stimulated conditions in three experimental systems. Synaptic profiles were sampled from stratum pyramidale and radiatum of the CA1 region from (1) perfusion fixed mouse brains, (2) immersion fixed rat organotypic slice cultures, and from (3) rat dissociated hippocampal cultures of mixed cell types. Synapses were stimulated in the brain by a 5 min delay in perfusion fixation to trigger an ischemia-like excitatory condition, and by treating the two culture systems with 90 mM high K^+^ for 2–3 min to depolarize the neurons. Upon such stimulation conditions, the presynaptic terminals of the inhibitory synapses exhibited similar structural changes to those seen in glutamatergic excitatory synapses, with depletion of synaptic vesicles, increase of clathrin-coated vesicles and appearance of synaptic spinules. However, in contrast to excitatory synapses, no structural differences were detected in the postsynaptic compartment of the inhibitory synapses upon stimulation. There were no changes in the appearance of material associated with the postsynaptic membrane or the length and curvature of the membrane. Also no change was detected in the labeling density of gephyrin, a GABAergic synaptic marker, lining the postsynaptic membrane. Furthermore, virtually all inhibitory synaptic clefts remained rigidly apposed, unlike in the case of excitatory synapses where ~ 20–30% of cleft edges were open upon stimulation, presumably to facilitate the clearance of neurotransmitters from the cleft. The fact that no open clefts were induced in inhibitory synapses upon stimulation suggests that inhibitory input may not need to be toned down under these conditions. On the other hand, similar to excitatory synapse, EGTA (a calcium chelator) induced open clefts in ~ 18% of inhibitory synaptic cleft edges, presumably disrupting similar calcium-dependent trans-synaptic bridges in both types of synapses.

**Supplementary Information:**

The online version contains supplementary material available at 10.1186/s13041-024-01151-0.

## Introduction

Mammalian hippocampus is well-studied for its relevance in learning and memory, which includes the integration of various excitatory and inhibitory inputs carried out by glutamatergic and GABAergic synapses, respectively [1]. Electron microscopy studies provide ultrastructural clues to suggest functional implications, yet compared to excitatory synapses, relatively few studies focused on inhibitory synapses [2]. Some studies reported changes in the number of inhibitory synapses upon chemical inhibitory long term depression in dissociated cell cultures [3], upon chemical long term potentiation (LTP) induction in slice cultures [4], and upon theta burst stimulation LTP in mature rat brain slices [5]. However, detailed ultrastructural characterization of GABAergic inhibitory synapse upon stimulation has been lacking.

Reported stimulation-induced structural changes in excitatory synapses, on the other hand, included gradual increase in thickness and curvature of the postsynaptic density (PSD) [6–8], redistribution of presynaptic proteins [9], rapid formation of spinules in synaptic terminals [10], and opening of synaptic clefts at edges of PSD [11]. The present study reexamined the same archived samples from above-mentioned studies where hippocampal dissociated and organotypic slice cultures were depolarized with high K^+^, and adult mouse brains were perfusion-fixed with a delay, to look for stimulation-induced structural changes in inhibitory synapses. We also carried out additional experiments in dissociated hippocampal neuronal cultures, using an antibody against gephyrin, a specific GABAergic synapse marker [2, 12], , to assess whether there is any change in its labeling density and to study synaptic cleft of inhibitory synapses upon depolarization and under calcium-free conditions.

## Methods

### Perfusion fixation of mouse brains

Samples from four perfusion-fixed mouse brains from a previously published report [6] were reexamined here for structural changes of inhibitory synapses. Briefly, adult male mice, 25–35 g in weight, were deeply anesthetized with isoflurane and perfusion fixed through the heart, first with 3.75% acrolein + 2% paraformaldehyde in PBS, then followed by 2% paraformaldehyde in PBS for two NIH Swiss mice (exp 1), or with 2% glutaraldehyde + 2% paraformaldehyde in 0.1 M sodium cacodylate buffer at pH7.4 for two C57 black mice (exp 2). The time interval starting from the moment the diaphragm was cut to the moment when the outflow from the atrium turned from blood to clear fixative was recorded. Those animals that were successfully perfused within 100 s were classified as “fast” perfusion. For the “delayed” perfusion experiments, calcium- and magnesium-containing PBS was first perfused through the heart for 5 min before the start of the fixative. Neurons were mostly under resting state after fast perfusion, while delayed perfusion fixation promoted an ischemic excitatory state [6]. The perfusion-fixed brains were dissected and vibratomed into 100 μm thick coronal slices and stored in 2% glutaraldehyde in 0.1 M cacodylate buffer at 4˚C.

### Preparation, treatments and fixation of rat hippocampal organotypic slice cultures

All samples were from a previous study [10] and reexamined here for structural changes of inhibitory synapses. Briefly, the hippocampus was removed from postnatal 6–8 day old rats and cut at 250 μm thickness with a tissue chopper. Slices were placed on a cell culture inserts in six-well culture dishes and used 10–14 days in vitro with the dishes on a floating platform in a water bath at 37 °C, treated with control medium or high K^+^ (90 mM) for 2–3 min. Samples were fixed with 2% glutaraldehyde and 2% paraformaldehyde, or 4% glutaraldehyde in 0.1 M cacodylate buffer at pH 7.4 for 1–3 h at room temperature and then stored at 4˚C.

### Preparation, treatments, fixation and pre-embedding immunogold labeling of rat dissociated hippocampal neuronal cultures

Some glutaraldehyde-fixed samples were from previously published reports [9, 11] and reexamined here for inhibitory synapses. Additional experiments were performed for the present study for immunogold labeling of gephyrin, a GABAergic synapse marker.

Briefly, cell cultures were prepared from embryonic 20-day-old rat fetuses by papain dissociation, and then plated on glial feeder cultures, and examined at 19–22 days in vitro (DIV). Cell culture dishes were placed on a floating platform in a water bath maintained at 37˚C. Control incubation medium was HEPES-based Krebs Ringer at pH 7.4. High K^+^ medium was at 90 mM KCl, with osmolarity compensated by reducing the concentration of NaCl. N-methyl-D-aspartic acid (NMDA) medium contained 60 µM NMDA in the control medium. EGTA, a calcium chelator, was at 1 mM in Ca^2+^-free medium, osmolarity compensated with sucrose. Cell cultures were washed with control medium and treated for (1) 2–3 min with control or high K^+^ medium, (2) 2 min with control or NMDA medium, and (3) 5 min with control or EGTA-medium, then were fixed immediately. Some cell cultures were fixed at 4–5 DIV [13, 14], and reexamined here for inhibitory synapses. For the present study, additional cell cultures were plated without a glial feeder layer, fixed at 5 DIV and labeled with an antibody against gephyrin.

For optimal structural preservation, cells were fixed with 4% glutaraldehyde in 0.1 M cacodylate buffer at pH 7.4 for 30 min to 1 h at room temperature and then stored in fixative at 4˚C. For pre-embedding immunogold labeling, cells were fixed with 4% paraformaldehyde in phosphate buffered saline (PBS) for 30–45 min at room temperature, then washed in PBS and stored in PBS at 4˚C. Samples for pre-embedding immunogold labeling [15] were permeabilized/blocked with 0.025-0.1% saponin/5% normal goat serum in PBS for 30 min, incubated with primary antibody for 1–2 h, incubated with secondary antibody conjugated to 1.4 nm gold particles (1:200, Nanogold from Nanoprobes, Yaphand, NY) for 1 h, washed in water and silver enhanced (HQ silver enhancement kit, Nanoprobes) to make the small gold particles visible. All steps were carried out at room temperature.

Mouse monoclonal antibody against gephyrin (1:100, clone mAb7a) and rabbit polyclonal antibody against Homer 1b/c (1:250) were from Synaptic Systems (Goettingen, Germany). Rabbit polyclonal antibody against synaptophysin (1:250) was from DAKO (Glostrup, Denmark). Mouse monoclonal antibodies against pan Shank (1:200, clone N23B/49) were from NeuroMab (Davis, CA). Guinea pig polyclonal antibody against Piccolo (1:100) was a gift from Dr. Eckart Gundelfinger (Leibniz Institute for Neurobiology, Magdeburg, Germany). Mouse monoclonal antibody against Bassoon (1:100, clone SAP7F407) was from Stressgen (Victoria, BC, Canada).

### Electron microscopy

Samples fixed with glutaraldehyde for structural analysis were post-fixed with 1% osmium tetroxide in 0.1 M cacodylate buffer for 1 h on ice and stained with 1% uranyl acetate in 0.1 N acetate buffer at pH 5.0 overnight at 4˚C. Samples for immunogold labeling were treated with 0.2% osmium tetroxide in 0.1 M phosphate buffer for 30 min on ice, followed by 0.25% uranyl acetate in acetate buffer at pH 5.0 at 4˚C for 30 min–1 h. Samples were then dehydrated in a graded series of ethanol and embedded in epoxy resins. Thin sections were cut at ~ 70 nm and counterstained with lead citrate. Images were photographed on a JEOL 1200 EX transmission electron microscope at 60 KV with a bottom-mounted digital CCD camera (AMT XR-100, Danvers, MA, USA).

### Morphometry

#### Identification of synapses and sampling criteria

Identification of synapses was based on criteria described in a classic EM atlas [16] and a review [17]. The present study focused on hippocampal GABAergic inhibitory synapses, which are characterized by (1) clusters of SV in presynaptic axonal terminals, (2) the synaptic cleft with rigid apposition between the pre- and postsynaptic membranes with a gap of ~ 20 nm, and (3) filamentous trans-cleft material in the synaptic cleft. Sections were screened at magnification of 10 K for presence of synapses, and then zoomed to magnification of 40 K for photography.

For perfusion-fixed brains [6] and organotypic hippocampal slice cultures [10], inhibitory synapses were sampled from the clusters of neuronal somas in stratum pyramidale and the proximal region (< 60 μm from stratum pyramidale) of stratum radiatum in the CA1 region. At these locations, virtually all inhibitory synapses were formed by parvalbumin expressing basket cells [18]. In contrast, dissociated hippocampal cultures contain a mixture of inhibitory synapses form all regions of the hippocampus, including CA1, CA2, CA3 and dentate gyrus, with different types of GABAergic synapses. Thus, measurements of synaptic vesicle depletion and number of clathrin-coated vesicles per presynaptic terminals as well as length and curvature of postsynaptic membrane were made from brains and slice cultures, where inhibitory synapses are from a single population. For comparison, length and curvature of postsynaptic membranes from gephyrin-labeled dissociated cell cultures were also measured. Additionally, images from distal region of stratum radiatum (> 175 μm of stratum pyramidale) where many inhibitory synapses are somatostatin positive were also sampled from perfusion-fixed brains for scoring of open cleft.

#### Measurement of synaptic vesicles sizes

The average diameter of a synaptic vesicle was determined by measuring its maximal diameter (L1) and a second diameter (L2) taken from the midpoint of L1 perpendicular to L1. The average diameter was defined as (L1 + L2)/2. Measurements were taken from the outside edge of the vesicle membrane.

#### Scoring of adherence junctions near inhibitory synapses

Adherence junctions (punctum adhaerens) [16] were identified by their characteristic features of patches of parallel plasma membranes between two cells, each with dense materials on the cytoplasmic side, and with a uniform distance between the two apposing membranes. The presence of adherence junctions was scored for every synaptic profile encountered, and a percentage of adherence junctions / total number of synaptic profiles in single sections was calculated.

#### Measurement of depletion of synaptic vesicles near active zones in presynaptic terminals of inhibitory synapses

We applied the same measurement criteria [9], to divide the presynaptic area within 200 nm of the active zone into three zones. Zones I and II are 33 nm wide each, and the two zones combined contain ~ two staggered rows of synaptic vesicles immediately adjacent to the presynaptic membrane, while Zone III contains synaptic vesicles further away (67–200 nm) from the presynaptic membrane.

#### Scoring of clathrin-coated pits (CCP) and vesicles (CCV) in presynaptic terminals

CCPs and CCVs in presynaptic terminals were identified by the structural characteristics of assembled clathrin coat [19]. The numbers of CCVs and CCPs were scored and pooled from every inhibitory presynaptic terminal profile encountered, and an average number per 100 presynaptic terminals was calculated for each sample [19].

#### Measurement of length and curvature of the postsynaptic membrane of inhibitory synapses

Measurement criteria were similar to those described previously [6]. Briefly, due to the lack of a prominent postsynaptic density in inhibitory synapses, the length of the postsynaptic membrane was only measured from perfectly cross-sectioned synaptic profiles where the rigidly apposed pre- and postsynaptic membranes with a ~ 20 nm cleft, containing trans-cleft filamentous material, can be discerned. Once the length of the postsynaptic membrane was determined, then the curvature of the postsynaptic membrane was calculated (the height divided by the length of the arc) into a percentage value. A curvature of zero denotes a flat postsynaptic membrane which does not arch up or down. Micrographs for measurement were oriented so that presynaptic terminals were consistently situated at the top of the postsynaptic membrane, with positive and negative values denote postsynaptic membranes that arch up or down, respectively.

#### Measurement of gephyrin labeling density at the postsynaptic membrane

Every gephyrin-labeled postsynaptic membrane was measured. Labeling density was calculated by counting the total number of silver enhanced particles of label for gephyrin, divided by the length of the postsynaptic membrane, and expressed as number of gold particles/µm.

#### Scoring of ‘open cleft’ at the lateral edges of the postsynaptic membrane of inhibitory synapses

**Fig. 1:**
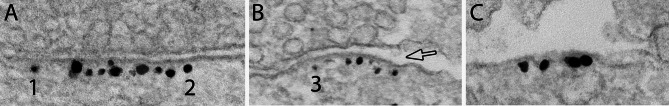
Scoring of synaptic cleft of inhibitory synapses as normal **(A)**, open **(B)** or unopposed **(C)**. Images from 3 weeks old-dissociated hippocampal cultures labeled for gephyrin, a GABAergic synapse marker, to delineate the postsynaptic membrane. If the pre- and post-synaptic membranes maintain the uniform gap at the edges of the synaptic cleft, those edges will be scored as “normal” (marked as “1” & “2” in **A**, and “3” in **B**). If the presynaptic membrane is separated from the postsynaptic membrane at the edge of the cleft, that edge will be scored as “open” (hollow arrow in **B**). If the gephyrin-labeled membrane is not apposed by a presynaptic terminal, it is scored as “unopposed” **(C)**. Scale bar = 100 nm

Each cross-sectioned, gephyrin-labeled inhibitory synapse was scored to classify the lateral edges of the synaptic cleft. A normal cleft is one with the pre- and postsynaptic membrane apposed with a uniform synaptic cleft (Fig. 1A). An “open” cleft is where the presynaptic membrane is separated from the gephyrin-labeled postsynaptic membrane (hollow arrow in Fig. 1B). an “unopposed” gephyrin-labeled patch of membrane is without an apposing presynaptic terminal (Fig. 1C).

### Statistical analysis

Mean values were evaluated by Student’s t-test between two groups, and by one-way ANOVA with Tukey’s post-test among three groups. Statistical significance on frequency of open clefts was evaluated by Chi-Square test of independence.

## Results

### Structural characterization of hippocampal inhibitory synapses under resting conditions

#### In stratum radiatum of CA1 region of mouse brains

**Fig. 2:**
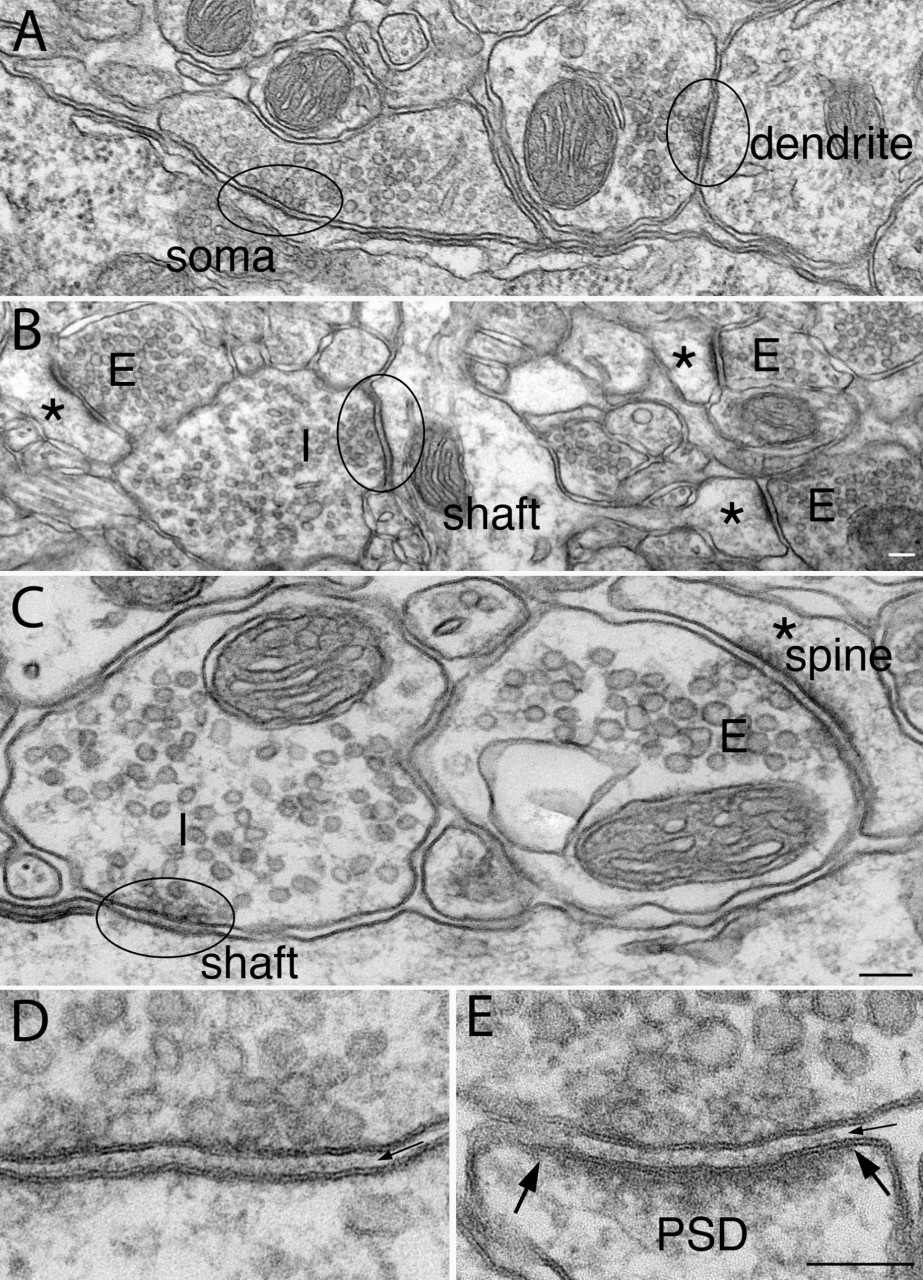
Representative images from stratum radiatum of the CA1 region of mouse hippocampus. Inhibitory synapses (enclosed in ellipses) are located on soma and dendritic shafts (**A**, **B**, **C**), while excitatory synapses (the presynaptic terminals of which are marked as “E”) are mostly on spines (asterisk in **B**, **C**) with a prominent postsynaptic density (PSD). Enlarged images of inhibitory **(D)** and excitatory **(E)** synapses showed similar width (~ 20 nm) in synaptic cleft, containing similar cleft material (small arrows in **D** & **E** point to synaptic cleft between uniformly apposed pre- and postsynaptic membranes). The size and shape of synaptic vesicles (SV) in inhibitory and excitatory synapses in **A** & **B** were similar (samples fixed with 2% glutaraldehyde and 2% paraformaldehyde), but SV size was smaller in inhibitory than in excitatory synapses in **C**- **E** (samples fixed with 3.5% acrolein followed by paraformaldehyde and glutaraldehyde). Scale bar = 100 nm

Consistent with previous EM reports [16], in adult mouse brain’s CA1 region of the hippocampus, the great majority of inhibitory synapses are on neuronal soma and dendritic shafts (Fig. 2A) while the great majority of excitatory synapses are on dendritic spines (asterisk in Fig. 2B). Aside from this difference in preferential location of the two types of synapses, the most conspicuous structural difference is that excitatory synapses have a prominent postsynaptic density (PSD, the edges of which are marked by two solid arrows in Fig. 2E), hence the two sides of the synaptic cleft appear “asymmetric” [16, 20]. In contrast, the inhibitory synapses lack a PSD, thus, appear “symmetric” with similar appearance in the pre- and postsynaptic compartments at the junctional area (ellipses in Fig. 2A -C and D). This difference in appearance of the two types of synapses are due to their different compositions in the postsynaptic scaffold proteins [12, 21].

Another structural difference is that synaptic vesicles (SV) were smaller in the GABAergic inhibitory synapses (Fig. 2D; ~35 nm in diameter) than in the glutamatergic excitatory synapses (Fig. 2E; ~45 mm in diameter) in samples initially fixed with acrolein (Fig. 2C-E). However, when samples were fixed with glutaraldehyde mixed with paraformaldehyde (Fig. 2A-B), both inhibitory and excitatory SVs showed similar diameters of ~ 45 nm. The present observation is in line with a previous EM study showing different processing methods affected the size and shape of SVs containing different transmitters [22]. Thus, size and shape of SVs can only be used to identity inhibitory vs. excitatory synapses under certain specific fixation and processing conditions.

Notably, the two types of synapses showed similar structural features at the synaptic cleft with a uniform distance of ~ 20 nm between the pre-and postsynaptic membranes, spanned with similar-looking cleft material (small arrows in Fig. 2D & E).

#### In rat organotypic slice cultures and dissociated cell cultures

**Fig. 3:**
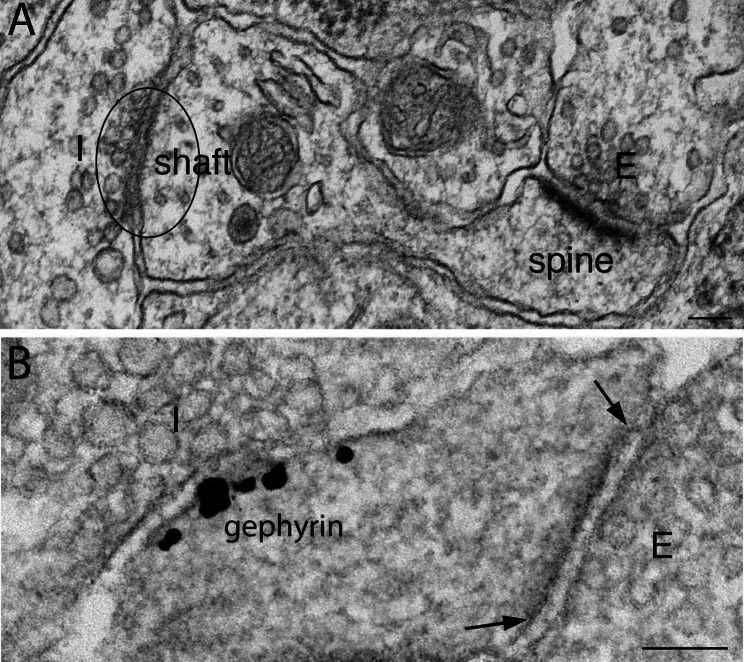
Examples of inhibitory synapses from hippocampal organotypic slice cultures **(A)** and dissociated cell cultures **(B)**. In slice cultures, inhibitory synapses (the presynaptic terminal of which was marked as “I”) were typically located on dendritic shaft, while excitatory synapses (marked as “E”) were on spines, with a distinctive PSD. **(B)** In 3 weeks old-dissociated hippocampal cell cultures, an inhibitory **(I)** synapse was labeled by a gephyrin antibody, a GABAergic synaptic marker. The same dendrite also exhibited an excitatory synapse **(E)** with a distinctive PSD (the edges of which marked by two arrows). Scale bar = 100 nm

Organotypic hippocampal slice cultures retain the anatomical organization and synaptic connection of the intact brains. Thus, the great majority of inhibitory synapses were located on the somas of stratum pyramidale and on dendritic shafts in stratum radiatum (Fig. 3A, marked as “I”), while the great majority of excitatory synapses were on spines (Fig. 3A, marked as “E”). However, in 3 weeks old-dissociated hippocampal cell cultures, many excitatory synapses are found on dendritic shafts (Fig. 3B) as well as on spines. The in vitro samples yielded synapses with similar structural features as their counterparts in brain, in that the excitatory synapses displayed distinctive PSDs. Although inhibitory synapses lack a prominent PSD, their postsynaptic membrane specifically label for gephyrin, a GABAergic synaptic marker [2] (Fig. 3B).

In the present study, all in vitro samples were fixed with 4% glutaraldehyde in 0.1 M cacodylate buffer for ultrastructural characterizations (Fig. 3A), and with 4% paraformaldehyde in PBS for pre-embedding immunogold labeling (Fig. 3B). Under these fixation and sample processing conditions, no conspicuous differences were detected between inhibitory and excitatory synapses in SV size, synaptic cleft width, or appearance of synaptic cleft materials.

#### Adherence junctions near inhibitory synapses

**Fig. 4:**
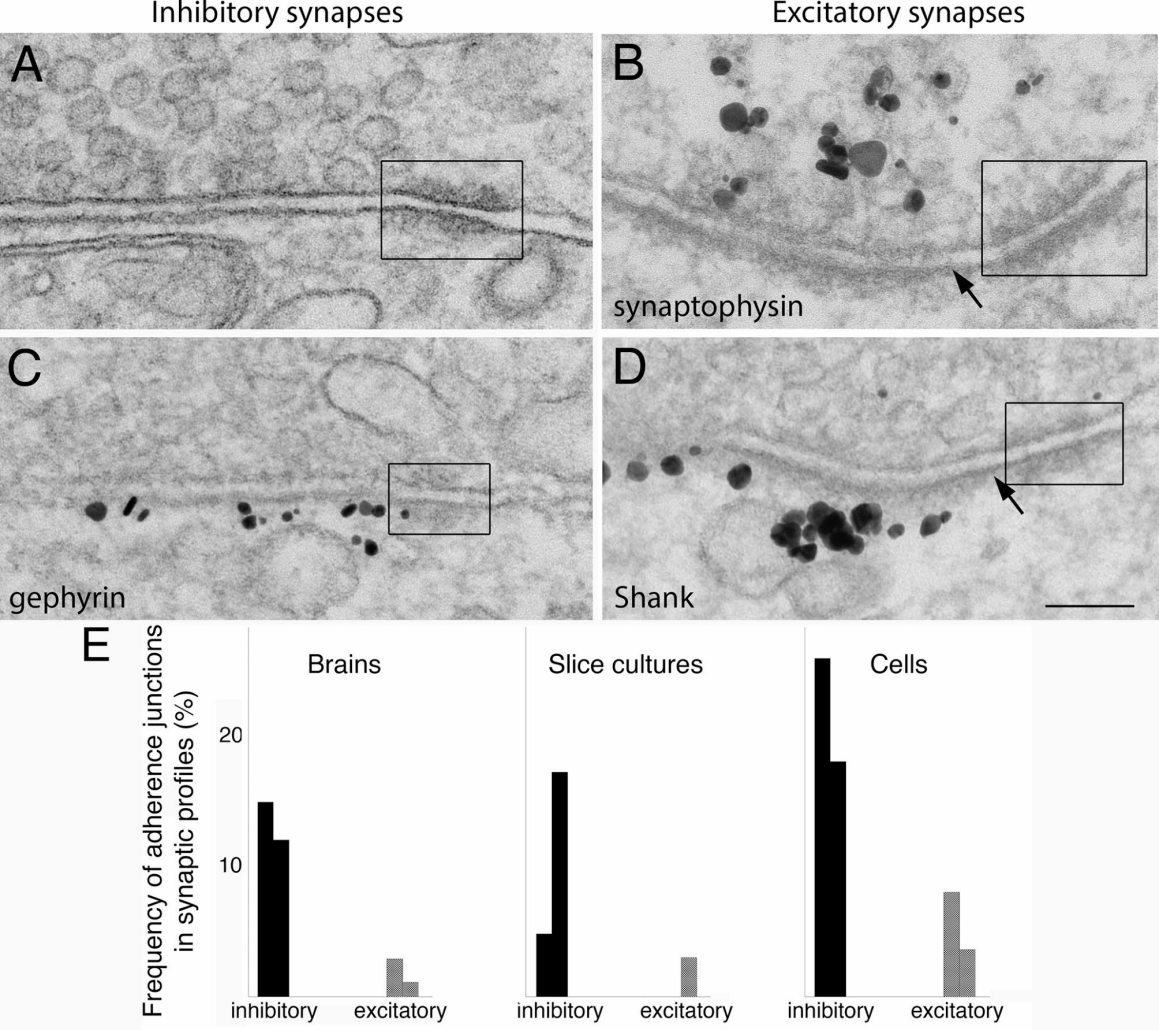
Adherence junctions (boxed area) were often seen adjacent to inhibitory synapses (**A**, **C**), and occasionally at excitatory synapses (**B**, **D**). All examples here were from 3 weeks old-dissociated cell cultures. The distance of the gap between the two membranes of the adherence junction was narrower than synaptic cleft in both type of synapses. Label for gephyrin **(C)**, a marker for GABAergic inhibitory synapse, clearly delineate the postsynaptic membrane next to the adherence junction. Label for synaptophysin **(B)**, a SV membrane protein [9], and label for Shank **(D)**, a PSD scaffold protein [7], specifically labeled the pre-and postsynaptic side of excitatory synapses. Scale bar = 100 nm. **(E)** Occurrence frequency (%) of adherence junctions in inhibitory vs. excitatory synaptic profiles from three experimental systems, 2 experiments each (Supplementary Material 1: Table S1)

We noticed that adherence junctions were more frequently encountered near inhibitory synapses (Fig. 4A, C) than near excitatory synapses (Fig. 4B, D). Quantification of occurrence frequencies of adherence junctions show a similar trend in all three experimental systems (Fig. 4E; Supplementary Material 1: Table S1).

Adherence junctions (boxed in Fig. 4) are composed of two rigidly apposed membranes between the pre- and postsynaptic membranes, each with dense material on their cytoplasmic side of the membranes, and with some trans-gap filamentous material between the apposing membranes. The distance of this gap was narrower (~ 15 nm) than that of the synaptic cleft (~ 20 nm), and this difference is likely due to the different composition of molecules at adherence junctions [23] vs. at synaptic clefts [24].

Due to the lack of a prominent PSD in inhibitory synapses, the nearby adherence junctions with characteristic dense material typically stood out distinctively (boxed in Fig. 4A). The juxtaposed location of adherence junctions (boxed in Fig. 4C) at inhibitory synapses is further illustrated by label for gephyrin of the inhibitory postsynaptic membrane. In contrast, in excitatory synapses, even though the prominent PSD clearly delineates the border of the synaptic contact (arrows in Fig. 4B, D), the adjacent adherence junction (boxed in Fig. 4B, D) could sometimes be misidentified as part of the PSD unless the presynaptic side of the adherence junction was unequivocally caught in thin sections (Fig. 4B), or the PSD was specifically immunolabeled while the adherence junction was not (Fig. 4D).

#### Inhibitory synapses were formed early in development

**Fig. 5:**
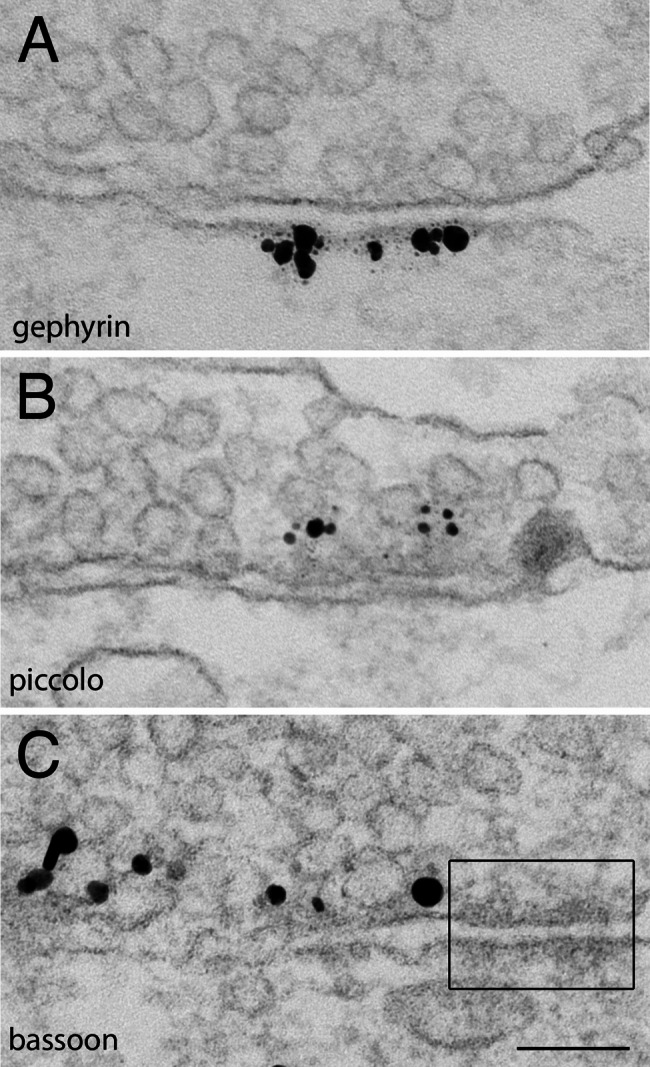
Inhibitory synapses in dissociated cultures at 4 **(C)** and 5 (**A**, **B**) days in culture. Label for gephyrin **(A)** marked the postsynaptic membrane, and label for piccolo **(B)** and bassoon **(C)** marked the presynaptic active zone. Adherence junction (box in C) was also present early in development. Scale bar = 100 nm

Similar to excitatory synapses [13, 14], inhibitory synapses were formed as early as 4 DIV in dissociated cultures. Inhibitory synapses in these young cultures were identified with immunolabeling of gephyrin at the postsynaptic membrane (Fig. 5A), or piccolo (Fig. 5B) and bassoon (Fig. 5C) at the presynaptic active zone [13]. Adherence junctions were also present near inhibitory synapses at this young age (boxed in Fig. 5C).

#### Structural changes of inhibitory synapses upon stimulation

In the present study, stimulation is carried out by a 5 min delay in perfusion fixation for brain tissues to cause an ischemia-like excitatory condition, and by depolarization with high K^+^ (90 mM for 2–3 min) in organotypic slice cultures and dissociated cell cultures, where high extracellular potassium concentration will generate action potentials in axons leading to synaptic vesicle release. This depolarization-induced SV release is expected in both the excitatory and inhibitory presynaptic terminals.

#### Structural changes at presynaptic terminals

##### Depletion of synaptic vesicles

**Fig. 6:**
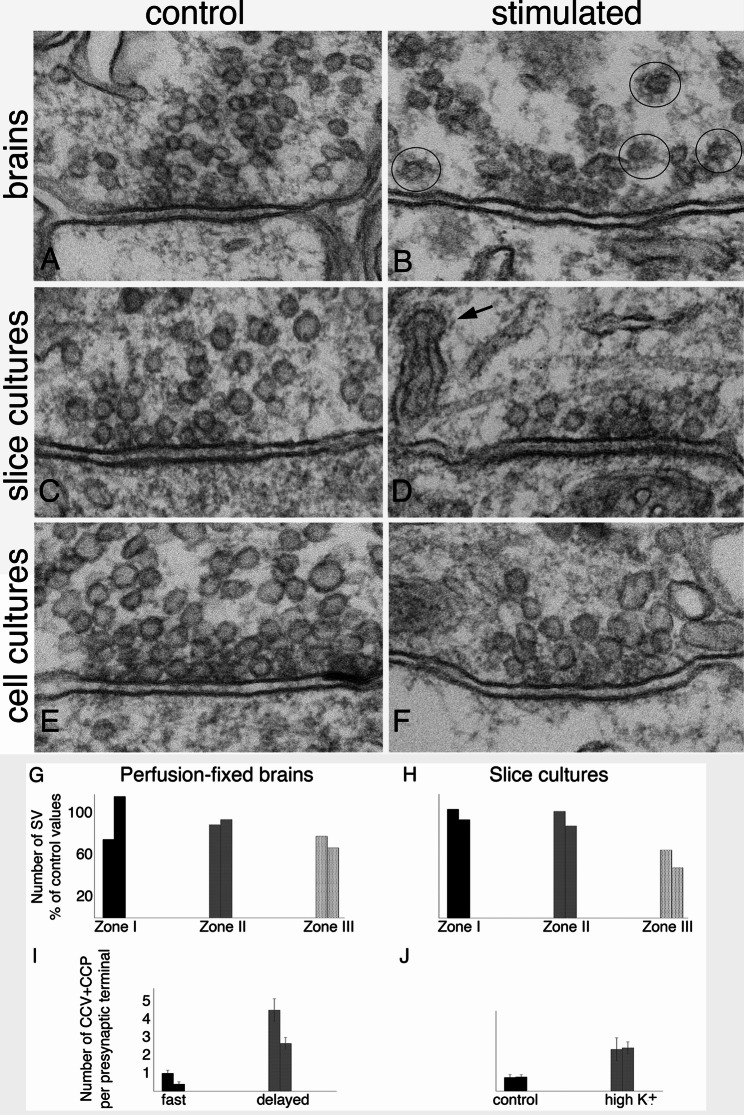
Inhibitory synapses under control vs. stimulated conditions sampled from stratum pyramidale and proximal stratum radiatum of the CA1 region of adult mouse hippocampus (**A**, fast perfusion; **B**, delayed perfusion; with initial acrolein fixation), organotypic slice cultures (**C**, control; **D**, depolarization with 90 mM K^+^ for 3 min), and from 3 weeks old-dissociation hippocampal cultures (**E**, control; **F**, depolarization with 90 mM K^+^ for 2 min). Upon stimulation, the presynaptic terminals showed SV depletion in all three experimental systems (left column vs. right column). (**G**, **H**) Bar graphs showing SV numbers upon stimulation as percent of control samples in zone I (0–33 nm), zone II (34–66 nm), and zone III (67–200 nm from presynaptic membrane) from presynaptic terminals sampled from perfusion-fixed brains **(G)** and slice cultures **(H)**, 2 experiments each (Supplementary Material 1: Table S2). Clathrin-coated vesicles (circled in **B**) and spinules (arrow in **D**) were prevalent in stimulated brains **(B)** and slice cultures **(D)**, respectively. (**I**, **J**) Bar graphs on number of clathrin-coated pits (CCP) and vesicles (CCV) in presynaptic terminals under resting and stimulated conditions sampled from perfusion-fixed brains **(I)** and slice cultures **(J)**, 2 experiments each (Supplementary Material 1: Table S3). No structural changes were detected in the postsynaptic compartment of inhibitory synapses upon stimulation. Scale bar = 100 nm

Similar to excitatory synapses [9], upon stimulation, there was conspicuous depletion of SVs in inhibitory synapses from brains (Fig. 6A vs. B), slice cultures (Fig. 6C vs. D) and 3wk-old dissociated cell cultures (Fig. 6E vs. F). In brains and organotypic slice cultures, sampling of inhibitory synapses was restricted to stratum pyramidale and proximal stratum radiatum, and SV numbers were counted from an area within 200 nm of the active zone [9]. There were no significant differences in the number of SV located within 66 nm of the active zone between control and stimulated samples, but significant decrease of SV in the area further away (67–200 nm) from the active zone. In two experiments each, SV numbers in this zone III in delayed fixed brains decreased to 67 and 78% of those in fast fixed brains (Fig. 6G); and to 49 and 65% of control values in slice cultures upon 2–3 min of depolarization with high K^+^ (Fig. 6H; Supplementary Material 1: Table S2).

##### Increase of clathrin-coated pits and vesicles

As in the case of excitatory synapses [19], number of clathrin-coated pits (CCP) and vesicles (CCV) in inhibitory presynaptic terminals of delayed fixed brains increased to about 4.5 to 6.7-fold of those in fast fixed samples (Fig. 6I). Likewise, upon 2–3 min of depolarization with high K^+^, number of CCP and CCV increased to about 3-fold of control levels in slice cultures (Fig. 6J; Supplementary Material 1: Table S3). Interestingly, spinules were frequently seen in inhibitory axon terminals upon stimulation (arrow in Fig. 6D), as in the case of excitatory synaptic terminals, indicative of synaptic activity [10].

#### No structural changes detected in the postsynaptic compartment of inhibitory synapses upon stimulation

##### No change in length and curvature of postsynaptic membrane

As in the case for excitatory synapses [6], the length of the postsynaptic membrane of inhibitory synapses did not change upon stimulation (Supplementary Material 1: Table S4). The average length of postsynaptic membrane of inhibitory synapses in adult mouse hippocampal CA1 stratum pyramidale and proximal stratum radiatum region at ~ 230 nm is in line with the size of area from en face view of rat hippocampal GABAergic synapses at ~ 0.05 µm^2^ [25].

In contrast to excitatory synapses [6], there was no consistent change in the curvature of the postsynaptic membrane of inhibitory synapses (Supplementary Material 1: Table S4). The majority of inhibitory synapses had a relatively flat curvature (Fig. 6). The two sets of perfusion-fixed brains showed a slight decrease in curvature index, but the differences did not reach statistical significance. In organotypic slice cultures, one experiment showed a significant decrease, but the second experiment showed a slight increase. In dissociated, gephyrin-labeled cell cultures, one experiment showed a significant increase, but a second experiment showed a slight decrease (Supplementary Material 1: Table S4).

##### No change in gephyrin labeling density

Labeling density for gephyrin in dissociated cell cultures were measured as number of gold particles per µm of postsynaptic membrane. No significant differences were detected between control and high K^+^-treated samples (Supplementary Material 1: Table S5).

##### Few open clefts were detected in inhibitory synapses upon stimulation

Unlike in excitatory synapses [11] where ~ 20–30% of synaptic cleft edges were open upon stimulation, very few open clefts were seen in inhibitory synapses. In perfusion-fixed brains, no open clefts were seen in inhibitory synapses sampled in stratum pyramidale and proximal stratum radiatum (Fig. 6A, B) formed by parvalbumin-positive interneurons. Sampling from distal region of stratum radiatum where many inhibitory synapses are formed by somatostatin-positive interneurons also yielded no open clefts. Thus, inhibitory synapses formed by these two types of interneurons do not form open clefts upon delayed perfusion fixation. Similarly, no open clefts were seen in inhibitory synapses from organotypic slice cultures (Fig. 6C, D), and from dissociated cultures (Fig. 6E, F) whether under resting or stimulated conditions.

**Fig. 7:**
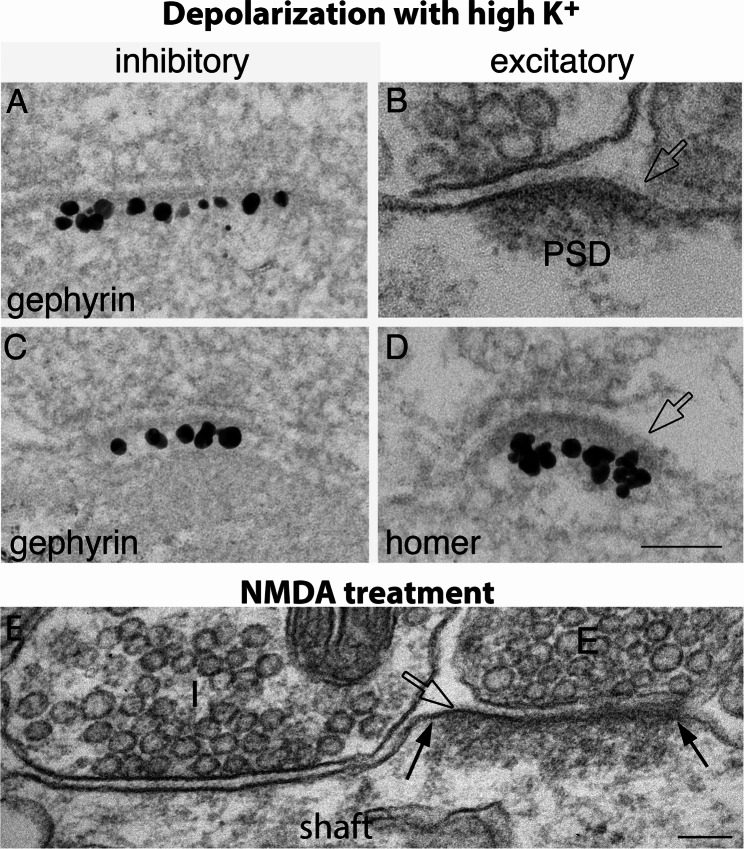
Inhibitory (**A**, **C**) and excitatory (**B**, **D**) synapses from 3 weeks old-dissociated hippocampal cultures upon 2–3 min depolarization with high K^+^, or NMDA treatment **(E)**. (**A**, **C**) Label for gephyrin underlie the postsynaptic membrane of GABAergic synapses and clearly delineates the edges of the synaptic cleft. Very few open clefts were present in inhibitory synapses upon depolarization, whereas the postsynaptic density (PSD, specifically labeled for homer in D; [7]) of excitatory synapses increased in thickness (**B**, **D**), and ~ 20% of the edges displayed open clefts (hollow arrows in B, D). (E) Activation of NMDA receptors (60 µM NMDA for 2 min) induced an increase in thickness of the PSD (the edges of which are marked with two solid arrows) and an open cleft (hollow arrow) in excitatory synapses (the presynaptic terminal marked as “E”). A nearby inhibitory synapse (marked as “I”) on the same dendritic shaft had normal cleft edges. Scale bar = 100 nm

Notably, images in Fig. 6 were sampled from acrolein- or glutaraldehyde-fixed materials, where inhibitory synapses may be overlooked due to the lack of a prominent postsynaptic membrane. In order to further verify that we did not miss any inhibitory synapses during sampling, we scored gephyrin-labeled inhibitory synapses for open clefts in 3 week-old dissociated cultures under different conditions (Supplementary Material 1: Table S6). Indeed, in contrast to excitatory synapses (Fig. 7B, D), the great majority of gephyrin-labeled inhibitory synapses did not have open cleft upon depolarization with high K^+^ (Fig. 7A, C), with only 1.5% (4 out of 261) cleft edges from 3 experiments showing open clefts. Similarly, 1.3% (3 out of 225) cleft edges under control conditions had open clefts. The finding that the stimulation-induced occurrence frequency of open clefts is higher in excitatory than in inhibitory synapses could have functional implications.

### Calcium affects structural integrity of synaptic cleft of inhibitory synapses

**Fig. 8:**
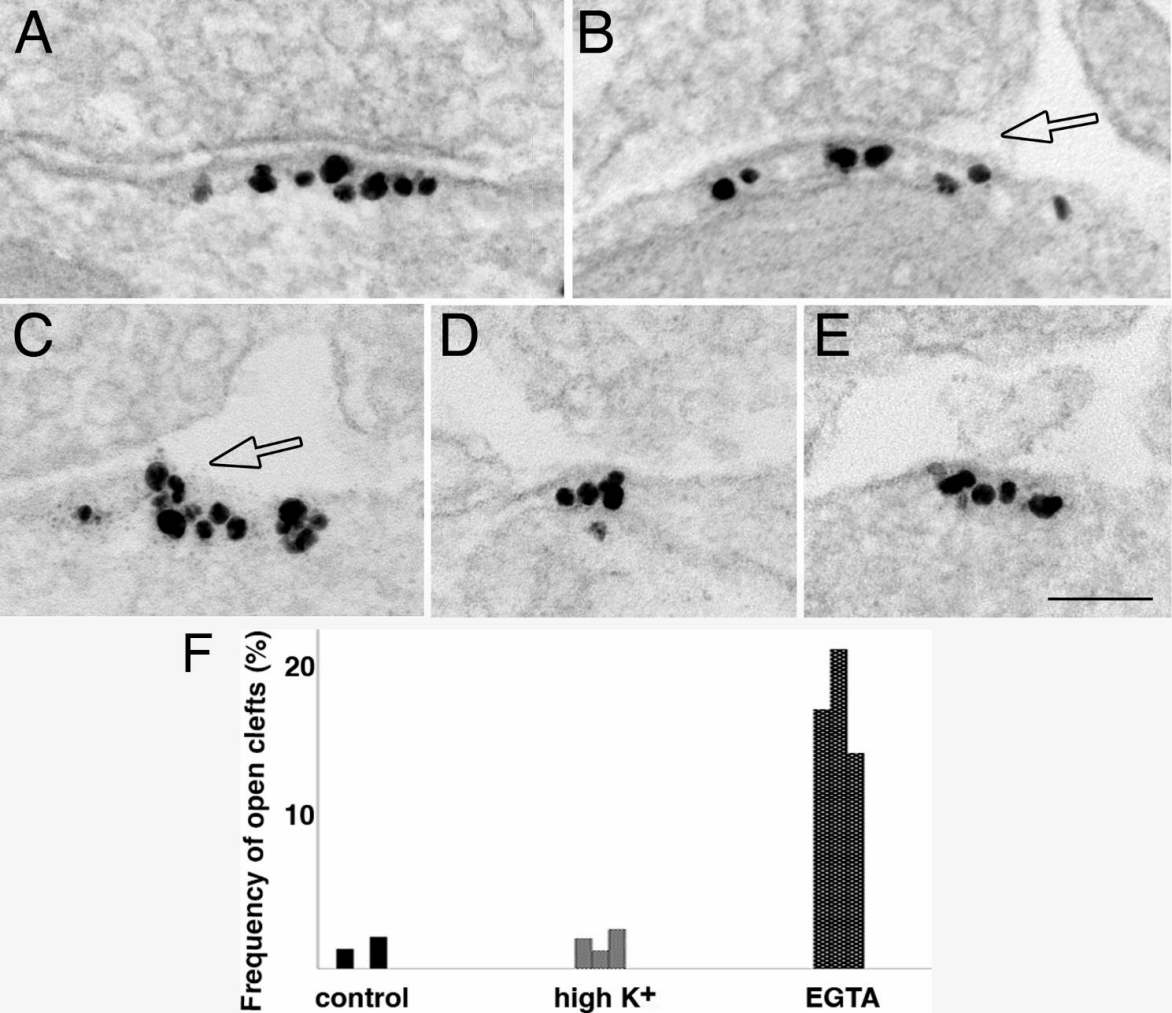
Synaptic clefts of Inhibitory synapses are disrupted under calcium-free conditions. Label for gephyrin, a GABAergic synapse marker, lined the cytoplasmic side of the inhibitory postsynaptic membrane. Images were from 3 week-old hippocampal dissociated cell cultures treated with 5 min of EGTA. **(A)** showed a synapse with normal clefts on both edges, while **B** & **C** showed open clefts (hollow arrows), and **D** & **E** showed unopposed patches of gephyrin-labeled membrane, sometimes with axon terminals nearby, but not close enough to be counted as a synaptic contact. Scale bar = 100 nm. **(F)** Bar graphs on occurrence frequency of open clefts in gephyrin-labeled inhibitory synapses under different conditions, 3 experiments (Supplementary Material 1: Table S6)

To test whether calcium affects the synaptic cleft integrity, 3 week-old dissociated cells were treated with a calcium chelator, EGTA (100 mM), for 5 min. In 3 experiments, about 82% (172 out of 209) of cleft edges from inhibitory synapses were normal (Fig. 8A) with the presynaptic membrane rigidly apposed to the gephyrin-labeled postsynaptic membrane with a uniform gap, while ~ 14–21% of inhibitory synaptic clefts were open (Fig. 8B, amp, C and F; Supplementary Material 1: Table S6) with the edges of the gephyrin-labeled patches of postsynaptic membrane exposed to the extracellular space separating from the presynaptic terminal (hollow arrows in Fig. 8B & C). Additionally, patches of gephyrin-labeled membranes were occasionally seen unopposed by presynaptic terminals (Fig. 8D, E), with 7, 4, and 2 unapposed patches in experiment 1, 2 and 3, respectively. Such unopposed gephyrin patches were only seen in EGTA-treated samples and not in control or high K^+^-treated samples. Thus, it appears that in some synapses, calcium-free conditions induced dissociation of trans-synaptic bridges between pre- and postsynaptic membranes, especially at the edges of the synaptic junction, and sometimes may proceed to complete separation of the junctional membranes.

## Discussion

The present EM study examined hippocampal GABAergic inhibitory synapses from three experimental systems: perfusion-fixed mouse brain, immersion-fixed organotypic slice cultures and dissociated cultures under different conditions to document changes in ultrastructure upon stimulation. These structural differences were also compared to those observed in glutamatergic excitatory synapses.

For excitatory synapses, translocation of many postsynaptic proteins upon stimulation has been documented [7]. For example, CaMKII, Shank, CYLD and IRSp53 are known to move toward the PSD upon stimulation, while SynGAP and AIDA moved away from the postsynaptic membrane. The redistribution of proteins at the PSD likely contributed to the appearance of thickened PSD. In contrast, we did not detect any changes in the thickness of associated material in the postsynaptic membrane of inhibitory synapses upon depolarization with high K^+^ for 2–3 min. There was also no change in the labeling density of gephyrin, a scaffold protein of GABAergic postsynaptic membrane. Future immunolabeling studies on redistribution of GABAergic postsynaptic proteins upon stimulation could further elucidate the organization of these proteins under resting and stimulated conditions.

The postsynaptic membrane of inhibitory synapses did not significantly arch up or down upon stimulation, unlike that of the excitatory synapses that consistently increased its curvature by arching into the presynaptic terminal [6, 8]. The different locations of the two types of synapses might have played a role in this difference: excitatory synapses are on spines, whose size and shape are known to change upon stimulation [26]; whereas inhibitory synapses are located on soma and dendrites where the postsynaptic membranes were situated among larger sheets of plasma membranes [25], and perhaps not as easily to change its curvature. However, the lack of detectible structural changes in the postsynaptic elements of inhibitory synapses does not mean that these synapses were not stimulated because their presynaptic counterparts showed clear signs of activation with depletion of SVs, increase of clathrin-coated vesicles and synaptic spinules.

Upon stimulation, approximately 20–30% of excitatory synaptic cleft edges were “open” from single thin section samplings. These open clefts are thought to facilitate the clearance of transmitters from the cleft under excitatory conditions, thus, may prevent damage from overstimulation [11]. The present study reexamined the same samples and found that open clefts were rare in inhibitory synapses upon stimulation, thus, probably no facilitation of clearing the inhibitory transmitter from the synaptic cleft. It is possible that maintaining the strength of inhibitory inputs this way may be helpful in toning down the effect of overstimulation.

Interestingly, EGTA treatment promotes the appearance of open clefts at 14–21% of cleft edges in inhibitory synapses, indicating the presence of similar calcium-dependent trans-synaptic bridges to excitatory synapses [11]. However, since synaptic activity does not promote cleft opening in inhibitory synapses as in excitatory synapses, the elements/mechanisms in excitatory synapse responsible for activity-induced disruption of trans-synaptic bridges are likely absent in inhibitory synapses.

One interesting feature of inhibitory synapses is that adherence junctions are often present nearby. The functional implications of a higher frequency of adherence junctions at inhibitory synapses than at excitatory synapses in hippocampal CA1 region is not clear. Assuming that adherence junctions provide structural stability [16], one possible explanation could be that excitatory synapses on spines undergo dynamic remodeling during synaptic activity [26], and thus, may need fewer adherence junctions to allow structural flexibility. In the present study, occurrence frequency of adherence junctions near excitatory synapses from single sections was scored at less than 3%, a number much lower than the 33% reported by a serial thin section study sampled from the same hippocampal CA1 region [27]. This difference is likely due to the much smaller area occupied by adherence junction compared to that of the adjacent PSD [27]. Hence, the chance of catching a small patch of adherence junction next to a larger PSD in a single thin section is much lower than that in a 3D reconstructed synapse in its entirety.

In conclusion, while inhibitory synapses exhibited activity-induced presynaptic changes, no conspicuous ultrastructural modifications were detected at the postsynaptic compartment. In contrast to excitatory synapses sampled from the same CA1 region of the hippocampus, synaptic cleft of GABAergic inhibitory synapses remained largely intact upon stimulation, perhaps to maintain the strength of inhibitory inputs. Both types of synapses showed similar partial opening of the cleft edges under calcium-free conditions, suggesting similar calcium-dependent trans-cleft bridges.

## Electronic supplementary material

Below is the link to the electronic supplementary material.

Supplementary Material 1

## Data Availability

The datasets generated and/or analyzed during the current study are available from the corresponding author on reasonable request.
