# Supplementary material for: Ultrastructural characterization of hippocampal inhibitory synapses under resting and stimulated conditions
Source: Mol Brain. 2024 Oct 22;17:76. doi: 10.1186/s13041-024-01151-0 (PMC11494804; doi:10.1186/s13041-024-01151-0)
Supplement: Supplementary file 1 — Supplementary Material 1 [file 13041_2024_1151_MOESM1_ESM.pdf]

Supplementary Table 1. Frequency of adherence junctions near inhibitory vs. excitatory synaptic profiles.

		Inhibitory synapses	Excitatory synapses
**Perfusion-fixed brains** (fast perfusion)	Exp 1	14.9% (47)	2.9% (34)<br>P<0.08
	Exp 2	12% (25)	1.1% (95)<br>P<0.01
**Organotypic slice cultures** (control conditions)	Exp 1	4.8% (21)	0 (31)<br>NS
	Exp 2	17.2% (29)	3.0% (33)<br>P<0.06
**Dissociated cell cultures** (control conditions)	Exp 1	25.9% (27)	8% (50)<br>NS
	Exp 2	18% (50)	3.6% (56)<br>P<0.05

(n) number of synaptic profiles scored.
For perfusion-fixed brains and organotypic slice cultures, sampling of synapses was from stratum pyramidale and proximal stratum radiatum of the CA1 region of hippocampus. For dissociated hippocampal cell cultures, sampling was from mixed cell types and samples were labeled for gephyrin.
Statistical significance within each experiment evaluated by Chi-Square test of independence. The trend that the occurrence frequency is higher in inhibitory than in excitatory synapses is consistent among 6 experiments, though only 2 reached statistical significance ($P<0.05$).

Supplementary Table 2. Percent changes (% of control values) of number of synaptic vesicles upon stimulation at different distances from the presynaptic membrane.

		**Distance from the presynaptic membrane**		
		**Zone I (0-33 nm)**	**Zone II (34-66nm)**	**Zone III (67-200 nm)**
**Perfusion-fixed brains (delayed/fast)**	**Exp1**	75% NS	89% NS	78% (P<0.05)
	**Exp 2**	116% NS	94% NS	67% (P<0.0001)
**Slice cultures (high K^+/control)**	**Exp 1**	104% NS	102% NS	65% (P<0.005)
	**Exp 2**	94% NS	88% NS	48% (P<0.0001)

Statistical analysis by Student's t-test between stimulated vs. control.

NS, not significant.

Supplementary Table 3. Number of clathrin-coated pits (CCP) and vesicles (CCV) in inhibitory presynaptic terminals under resting and stimulated conditions.

Conditions			Number of CCP + CCV	Number of terminals	Average number of CCP + CCV per 100 terminals, (% control value)
**Perfusion-fixed brains**	**Exp 1**	Fast	48	48	100 ± 17
		Delayed	113	25	452 ± 64 (452%) (P<0.0001)
	**Exp 2**	Fast	10	25	40 ± 13
		Delayed	80	30	267 ± 33 (668%) (P<0.0001)
**Slice cultures**	**Exp 1**	Control	16	21	76 ± 15
		2 min high K^+	42	18	233 ± 35 (307%) (P<0.0005)
	**Exp 2**	Control	30	38	79 ± 14
		3 min high K^+	53	22	241 ± 27 (305%) (P<0.0001)

Statistical analysis by Student's t-test between control vs. stimulated.

Supplementary Table 4. Length and curvature of the postsynaptic membrane in inhibitory synapses under resting vs. stimulated conditions.

Conditions			Length (nm)	Curvature index
**Perfusion-fixed brains**	**Exp 1**	Fast	244 ± 11 (47)	0.7 ± 0.9
		Delayed	212 ± 21 (25)<br>NS vs. fast	-0.2 ± 1.2<br>NS vs. fast
	**Exp 2**	Fast	226 ± 17 (25)	-0.8 ± 1.1
		Delayed	231 ± 15 (29)<br>NS vs. fast	-3.8 ± 1.0<br>NS vs. fast
**Slice cultures**	**Exp 1**	Control	313 ± 33 (21)	2.5 ± 1.2
		2 min high K^+	271 ± 25 (20)<br>NS vs. control	-2.4 ± 0.7<br>$P<0.001$ vs. control
	**Exp 2**	Control	266 ± 18 (31)	1.1 ± 1.0
		3 min high K^+	278 ± 27 (13)<br>NS vs. control	3.9 ± 1.3<br>NS vs. control
**Cell cultures**	**Exp 1**	Control	245 ± 18 (25)	2.1 ± 0.7
		3 min high K^+	238 ± 18 (41)<br>NS vs. control	5.4 ± 1.2<br>$P<0.05$ vs. control
	**Exp 2**	Control	263 ± 24 (36)	4.0 ± 0.8
		3 min high K^+	324 ± 30 (31)<br>NS vs. control	2.5 ± 0.8<br>NS vs. control

(n) – number of synaptic profiles sampled.

For perfusion-fixed brains and organotypic slice cultures, sampling of synapses was from stratum pyramidale and proximal stratum radiatum of the CA1 region of hippocampus. For dissociated hippocampal cell cultures, sampling was from mixed cell types and samples were labeled for gephyrin.

Statistical significance within each experiment evaluated by Student's t-test.

NS – not significant

Supplementary Table 5. Labeling density of gephyrin (number of gold particles/μm) at postsynaptic membrane of inhibitory synapses in dissociated hippocampal cultures under different conditions.

	**Control**	**High K^+**	**EGTA**
**Exp 1**	37.4 ± 2.8 (25)	37.3 ± 1.9 (41)	41.0 ± 2.1 (34)
**Exp 2**	43.6 ± 3.3 (18)	39.8 ± 2.5 (29)	42.7 ± 2.0. (30)
**Exp 3**	35.1 ± 1.9 (36)	39.9 ± 3.4 (31)	39.7 ± 2.8 (28)

(n) number of synaptic profiles sampled.
No statistical differences among conditions (by one way ANOVA) in all three experiments.

Supplementary Table 6. Percentage (%) of open cleft scored from gephyrin-labeled inhibitory synapses of dissociated hippocampal cell cultures under different conditions.

	**Control**	**3-5 min of High K^+**	**5 min EGTA**
**Exp 1**	1.3% (78)	2.0% (100)<br>NS vs. control	17.2% (87)<br>P<0.001 vs. control<br>P<0.0005 vs high K^+
**Exp 2**	0 (50)	1.2% (83)<br>NS vs. control	21.2% (66)<br>P<0.001 vs. control<br>P<0.0001 vs high K^+
**Exp 3**	2.1% (97)	2.6% (78)<br>NS vs. control	14.3% (56)<br>P<0.005 vs. control<br>P<0.05 vs high K^+

(n = total number of synaptic cleft edges scored)

Statistical significance within each experiment evaluated by Chi-Square test of independence.
